# Should patients undergo ascending aortic replacement with concomitant cardiac surgery?

**DOI:** 10.5830/CVJA-2016-026

**Published:** 2016

**Authors:** Mihriban Yalcin, Kaptan Derya, Melih Urkmez

**Affiliations:** Cardiovascular Surgery Department, Ordu State Hospital, Ordu, Turkey; Cardiovascular Surgery Department, Ordu State Hospital, Ordu, Turkey; Cardiovascular Surgery Department, Ordu State Hospital, Ordu, Turkey

**Keywords:** ascending aortic aneurysm, ascending aortic replacement, concomitant cardiac surgery, mortality

## Abstract

**Aim:**

To determine whether concomitant surgery is a predictor of mortality in patients undergoing surgery for ascending aortic aneursym.

**Methods:**

Ninety-nine patients who underwent ascending aortic aneursym surgery between January 2010 and January 2015 were included in this study. Nineteen patients underwent ascending aortic replacement (RAA) only, 36 underwent aortic valve replacement (AVR) and RAA, 25 underwent coronary artery bypass grafting (CABG) and RAA, 11 underwent the Bentall procedure, and eight underwent AVR, CABG and RAA.

**Results:**

Depending on the concomitant surgery performed with RAA, the mortality risk increased 2.25-fold for AVR, 4.5-fold for CABG, 10.8-fold for AVR + CABG and four-fold for the Bentall procedure, compared with RAA alone.

**Conclusion:**

Concomitant cardiac surgery increased the mortality risk in patients undergoing RAA, but the difference was not statisticaly significant. Based on these study results, patients undergoing cardiac surgery, with a pre-operative ascending aortic diameter of over 45 mm, should undergo concomitant RAA.

## Aim

Ascending aortic dilatation is the most common cardiac condition requiring surgery. Dilatation causes aneursym, dissection and rupture, and aortic valve insufficiency.1 The aorta is aneurysmatic when there is a 50% increase in its normal diameter. Underlying physiological circumstances, body surface area and the patient’s age are the main determinants of the size of the ascending aorta.^2^

Valvular or coronary cardiac diseases frequently occur with ascending aortic aneurysm. Indications for surgery depend on the underlying pathology or when dissection or rupture occur. Although there is weak evidence, the current ACC/AHA guidelines recommend concomitant replacement of a significantly enlarged ascending aorta at the time of cardiac surgery.^3^

Many other factors, including patient age, co-morbidities, type of valve prosthesis and surgeon-specific results must be considered when determining whether or not to replace the aorta.^4^ If the aortic valve leaflets, the annulus and the sinuses of Valsalva are intact, the aneurysm is replaced with a simple supracoronary Dacron tube graft. If the aortic valve is diseased but the aortic sinuses and annulus are normal, then a Wheat procedure [aortic valve replacement (AVR) and a separate ascending aortic replacement (RAA)] are performed. In cases with root dilation, a modified Bentall procedure (replacement of a vascular tube graft with a biological or mechanical aortic valve prosthesis and re-insertion of the graft into the coronary ostia) is the gold standard.

This retrospective study aimed to establish the effect of concomitant cardiac surgeries on mortality rates in patients undergoing ascending aorta surgery.

## Methods

Between January 2010 and January 2015, 99 patients underwent surgery for RAA at the Ordu State Hospital. We retrospectively reviewed the medical records of these patients. Those who underwent either mitral valve replacement (MVR) or any other cardiac surgery, or patients with aortic dissection were excluded. Information about age, gender, ejection fraction (EF), diabetes mellitus (DM), chronic obstructive pulmonary disease (COPD) and peripheral arterial disease (PAD) was collected.

The diameter of the ascending aorta, aortic cross-clamp time, cardiopulmonary bypass time, total circulatory arrest time, the type of cardiac surgery that was performed, use of intra-aortic balloon pump (IABP) support, and whether or not positive inotropic support was required were also evaluated. Mechanical ventilation time, total drainage amount, duration of intensive care and hospital stay, and death were evaluated (Tables 1,2).

**Table 1:** Descriptive statistics (mean ± standard deviation) of the examined variables for mortality

**	*Mortality*	
*Variables*	*No*	*Yes*
Age (year)	64.3 ± 8.46	70.6 ± 9.98
Aortic diameter (mm)	52.4 ± 5.31	51.8 ± 6.79
EF (%)	55.8 ± 9.75	47.6 ± 11.62
Cross-clamping time (min)	99.3 ± 41.16	139.1 ± 61.26
CPB time (min)	165.3 ± 59.83	193.8 ± 65.32
TCA time (min)	25.6 ± 7.34	29.2 ± 7.05
ICU stay (days)	2.65 ± 1.31	10.0 ± 14.03
Bleeding (ml)	501.8 ± 344.57	725.0 ± 381.09

CBP: cardiopulmonary bypass, EF: ejection fraction, ICU: intensive care unit , TCA: total circulatuar arrest.

**Table 2:** Frequencies and percentages [n (%)] of the examined variables for mortality

*Variables*	*Mortality*	
	No	Yes
COPD		
No	76 (85.4)	13 (14.6)
Yes	8 (80.0)	2 (20.0)
PAD		
No	80 (85.1)	14 (14.9)
Yes	4 (80.0)	1 (20.0)
DM		
No	65 (87.8)	9 (12.2)
Yes	19 (76.0)	6 (24.0)
Inotrope use		
No	41 (97.6)	1 (2.4)
Yes	43 (75.4)	14 (24.6)
IABP		
No	80 (89.9)	9 (10.1)
Yes	4 (40.0)	6 (60.0)

COPD: chronic obstructive pulmonary disease, DM: diabetes mellitus, PAD: peripheral arterial disease, IABP: intra-aortic balloon pump.

There were 39 female and 60 male patients. The mean size of the ascending aorta in our patients was 52.28 ± 5.78 mm in females and 52.30 ± 5.36 mm in males. The mean age was 65.23 ± 8.49 years for females and 65.05 ± 9.27 years for males.

The decision to replace the ascending aorta was based on the aortic diameter. Patients with an ascending aortic diameter of more than 45 mm mostly underwent concomitant RAA. In our study, 80 of 99 patients underwent multiple operations. Nineteen patients underwent RAA only, 36 underwent AVR and RAA, 25 underwent coronary artery bypass grafting (CABG) and RAA, 11 underwent a Bentall procedure, and eight patients underwent AVR, CABG and RAA.

AVR + RAA was performed in patients who had aortic valve stenosis or aortic valve regurgitance and a dilated ascending aorta with normal aortic sinuses. CABG + RAA was performed in patients who had coronary artery disease (CAD; coronary artery stenosis ≥ 70%). The Bentall procedure was performed in patients who had aortic root aneurysm. If the ascending aorta was dilated and the aortic root was normal, we replaced the aortic valve and the supracoronary ascending aorta. If the non-coronary sinus of valsalva was dilated, we replaced only the non-coronary sinus by tailoring the supracoronary graft to extend down to the aortic annulus, but the left and right sinuses and the coronary arteries were left intact. For patients with an aortic root abnormality and a dilated ascending aorta, the Bentall procedure is appropriate.

All surgeries were performed via a median sternotomy incision. Cardiopulmonary bypass (CPB) was established via right axillary cannulation and a single venous cannula, and antegrade and retrograde blood cardioplegia was performed. All ascending replacements were performed under deep hypothermic circulatory arrest with a nasopharyngeal temperature of 18 ± 1°C. Antegrade cerebral perfusion was used in all patients. Replacement of the ascending aorta was performed using a woven Dacron prosthetic graft (AlboGraft LeMaitre Vascular). If concomitant surgical procedures were required, distal coronary anastomosis and concomitant surgical procedures were performed before the replacement of the ascending aorta.

The proximal anastomosis was performed during aortic crossclamping and the distal anostomosis was performed under deeep hypothermic circulatory arrest. After appropriate blood pressure and cardiovascular stability were ensured, CPB was ended. Patients were taken to the intensive care unit (ICU) during the postoperative period.

## Statistical analysis

Statistical analysis was performed using SPSS 17.0 for Windows software (SPSS Inc, Chicago, IL, USA). Data are presented as the mean ± standard deviation for the numerical variables (e.g. age, diameter of ascending aorta) or as the number and percentage of cases for categorical variables (e.g. mortality, COPD, DM). Univariate logistic regression analysis was performed to assess the main factors associated with mortality. Variables in univariate analysis that were associated with mortality [p < 0.20 in the likelihood ratio test (−2LL)] were selected for multivariate logistic regression analyses.

All identified individual variables were analysed using a manual backward elimination procedure, starting with a full multivariate logistic regression model. Variables were kept in the model if the −2LL ratio test of the model with and without the variable was significant (p < 0.05). The odds ratios (OR) are presented with 95% confidence intervals (95% CI). The final individual model was tested using the Hosmer–Lemeshow test for goodness-of-fit

## Results

In the patients who died, age, ascending aortic diameter, crossclamp time, CPB time, total circulatory arrest time and ICU stay were longer, bleeding was greater and EF was lower than the patients who survived (Table 1). The extubation time was 17.2 ± 6.13 hours and patients were discharged after 7.30 ± 2.41 days. Patients who died had COPD (20%), PAD (20%) and DM (24%), and required inotrope use (24.6%) and IABP support (60%) ( Table 2).

Table 3 shows descriptive statistics for patients who underwent RAA alone and additional surgery. Patients who underwent AVR + CABG were older (71.3 ± 12.74 years) than those in the other groups. The ascending aortic diameter was larger in patients who underwent RAA alone (56.2 ± 4.03 mm) compared to the other groups. CPB time and extubation time in patients who underwent the Bentall procedure (229.5 ± 82.79 minutes, 15.6 ± 11.36 hours, respectively) were longer and there was more bleeding (716.7 ± 557.9 ml) compared to the other patients.

**Table 3:** Descriptive statistics (mean ± standard deviation) of the examined variables for additional operations

**	*Additional operations*				
*Variables*	*No*	*AVR*	*CABG*	*AVR+CABG*	*Bentall*
Age (years)	64.5 ± 8.68	64.3 ± 8.17	67.7 ± 5.65	71.3 ± 12.74	58.7 ± 11.36
Aortic diameter (mm)	56.2 ± 4.03	51.5 ± 5.59	49.6 ± 3.57	52.3 ± 7.85	54.3 ± 5.48
Cross-clamping time (min)	80.9 ± 48.46	97.2 ± 34	104.0 ± 29.43	167.8 ± 63.04	130.7 ± 53.87
TCA time (min)	26.6 ± 4.88	24.6 ± 8.10	29.4 ± 8.70	22.6 ± 3.50	25.5 ± 5.07
EF (%)	58.6 ± 8.42	55.4 ± 11.78	54.0 ± 9.81	50.4 ± 11.96	52.1 ± 4.88
CPB time (min)	144.0 ± 54.52	164.5 ± 44.28	159.6 ± 59.21	202.3 ± 61.6	229.5 ± 82.79
Extubation time (hour)	17.4 ± 4.41	15.2 ± 5.12	16.9 ± 9.21	11.4 ± 10.47	15.6 ± 11.36
ICU stay (day)	2.11 ± 0.46	4.06 ± 7.10	5.08 ± 8.04	2.5 ± 2.07	3.36 ± 2.80
Bleeding (ml)	373.7 ± 175.1	533.8 ± 355.7	541.3 ± 259.7	700.0 ± 539.3	716.7 ± 557.9
Discharge from hospital	6.89 ± 1.79	6.08 ± 2.98	6.04 ± 3.48	4.57 ± 4.5	8 ± 4.82

CBP: cardiopulmonary bypass, EF: ejection fraction, ICU: intensive care unit, TCA: total circulatory arrest.

Table 4 shows that mortality was 5.3% in patients who underwent RAA alone, 11.1% in patients who underwent AVR, 20% in patients who underwent CABG, 37.5% in patients who underwent AVR + CABG, and 18.2% in patients who underwent the Bentall procedure. The most frequently observed co-morbidities were DM (48%) in patients who underwent CABG, COPD (12.5%) in patients who underwent AVR + CABG, and PAD (25%) in patients who underwent AVR + CABG. All patients (100%) who underwent the Bentall procedure required inotrope, while 25% of patients who underwent AVR + CABG required IABP support.

**Table 4:** Frequencies and percentages [n (%)] of the examined variables for additional operations

**					
*Variables*	*No*	*AVR*	*CABG*	*AVR + CABG*	*Bentall*
Mortality					
No	18 (94.7)	32 (88.9)	20 (80.0)	5 (62.5)	9 (81.8)
Yes	1 (5.3)	4 (11.1)	5 (20.0)	3 (37.5)	2 (18.2)
COPD					
No	17 (89.5)	35 (94.6)	22 (88.0)	7 (87.5)	9 (90.0)
Yes	2 (10.5)	2 (5.4)	3 (12.0)	1 (12.5)	2 (10.0)
PAD					
No	16 (84.2)	37 (100.0)	25 (100.0)	6 (75.0)	11 (100.0)
Yes	3 (15.8)	0 (0.0)	0 (0.0)	2 (25.0)	0 (0.0)
DM					
No	17 (89.5)	33 (89.2)	13 (52.0)	6 (75.0)	6 (54.5)
Yes	2 (10.5)	4 (10.8)	12 (48.0)	2 (25.0)	5 (45.5)
Inotrope use					
No	10 (52.6)	22 (61.1)	9 (36.0)	1 (12.5)	0 (0.0)
Yes	9 (47.4)	14 (38.9)	16 (64.0)	7 (87.5)	11 (100.0)
IABP					
No	19 (100.0)	34 (94.4)	21 (84.0)	6 (75.0)	9 (81.8)
Yes	0 (0.00)	2 (5.6)	4 (16.0)	2 (25.0)	2 (18.2)

COPD: chronic obstructive pulmonary disease, DM: diabetes mellitus, PAD: peripheral arterial disease, IABP: intra-aortic balloon pump.

Univariate logistic regression analysis showed that the mortality risk was increased 2.321-fold in patients ≥ 70 years of age and 1.36-fold in men. The mortality risk was increased 2.25-fold in patients requiring RAA + AVR, 4.5-fold in patients requiring RAA + CABG, 10.8-fold in patients requiring RAA + AVR + CABG, and four-fold in patients requiring the Bentall procedure.

Patients with COPD were at 1.462 times higher risk of mortality, those with PAD were at 1.429 times higher risk and those with DM were at 2.281 times higher risk. Patients requiring inotropic drugs were at 13.329 times higher risk and those requiring IABP were at 13.333 times higher risk of mortality (Table 5).

**Table 5:** Potential risk factors associated with mortality in the univariate logistic regression equation

**	**	**	*Prevalence*	**	**	**	**	**	*95% CI for exp (B)*	
*Variable*	*No*	*Total no*	*(%)*	*B*	*SE*	*Walad*	*Sig*	*Exp (B)*	*Lower*	*Upper*
Constant				–1.72	0.280	37.774	<0.001	0.179		
Age (years)										
< 70	8	69	11.6							
≥ 70	7	30	23.3	0.842	0.572	2.162	0.141	2.321	0.756	7.127
Gender										
Female	5	39	12.8							
Male	10	60	16.7	0.307	0.591	0.271	0.603	1.360	0.427	4.332
Additional operations										
No	1	19	5.3			4.815	0.307			
AVR	4	36	11.1	0.811	1.156	0.492	0.483	2.250	0.233	21.694
CABG	5	25	20.0	1.504	1.143	1.733	0.188	4.500	0.479	42.248
AVR+CABG	3	8	37.5	2.380	1.261	3.564	0.059	10.800	0.913	127.754
Bentall	2	11	18.2	1.386	1.291	1.153	0.283	4.000	0.319	50.229
COPD										
No	13	89	14.6							
Yes	2	10	20.0	0.379	0.846	0.201	0.654	1.462	0.279	7.667
PAD										
No	14	94	14.9							
Yes	1	5	20.0	0.357	1.155	0.095	0.757	1.429	0.149	13.741
DM										
No	9	74	12.2							
Yes	6	25	24.0	0.824	0.588	1.966	0.161	2.281	0.720	7.221
Inotrope use										
No	1	42	2.4							
Yes	14	57	24.6	2.591	1.058	6.001	0.014	13.349	1.679	106.145
IABP										
No	9	89	10.1							
Yes	6	10	60.0	2.590	0.735	12.419	< 0.001	13.333	3.157	3.157

AVR: aortic valve replacement, CABG: coronary arterial bypass grafting, COPD: chronic obstructive pulmonary disease, DM: diabetes mellitus, PAD: peripheral arterial disease, IABP: intra-aortic balloon pump.

Multivariate logistic regression analysis final model results showed that the mortality risk was 9.779-fold higher in patients who required inotropic drugs and 9.029-fold higher in patients who required IABP compared to those who did not (Table 6).

**Table 6:** Potential risk factors associated with mortality in the multivariate logistic regression equation

**	**	**	**	**	**	*95% CI for exp (B)*	
*Variables*	*B*	*SE*	*Wald*	*Sig*	*Exp (B)*	*Lower*	*Upper*
STEP 1a (beginning model)							
Constant	–5.23	1.587	10.844	0.001	0.005		
Age (≥ 70 years)	1.146	0.732	2.452	0.117	3.144	0.750	13.190
Additional operations							
AVR	0.945	1.246	0.575	0.448	2.573	0.224	29.606
CABG	0.540	1.272	0.180	0.671	1.716	0.142	20.752
AVR+CABG	1.013	1.434	0.499	0.480	2.755	0.166	45.761
Bentall	0.088	1.426	0.004	0.951	1.092	0.067	17.856
DM (yes)	0.573	0.767	0.559	0.455	1.774	0.395	7.971
Inotrope use (yes)	2.510	1.134	4.899	0.027	12.308	1.333	113.645
IABP	2.173	0.881	6.091	0.014	8.789	1.564	49.379
STEP 4 (final model)							
Constant	–3.87	1.028	14.154	< 0.001	0.021		
Inotrope use (yes)	2.280	1.078	4.471	0.034	9.779	1.181	80.947
IABP (yes)	2.200	0.770	8.177	0.004	9.029	1.998	40.797

aVariable(s) entered on step 1: age, additional operations, DM, inotrope use, ABP.

AVR: aortic valve replacement, CABG: coronary arterial bypass grefting COPD: chronic obstructive pulmonary disease, DM: diabetes mellitus, PAD: peripheral arterial disease, IABP: intra-aortic balloon pump.

Five variables were included in the initial multivariate logistic regression model and three variables (gender, COPD, PAD) were excluded, based on the likelihood ratio test (p < 0.20). In the final model, the following were identified as being associated with mortality: inotrope (OR, 9.779) and IABP (OR, 9.029) (Table 6). The final model fit was tested using the Hosmer–Lemeshow test. The H–L statistic had a significance of 0.889, which means that it was not statistically significant and therefore our model was a good fit.

## Discussion

Ascending aortic aneurysms start from the aortic valve and extend to the innominate artery, and they generally require openheart surgery. Increasing age, hypertension, smoking, genetics, atherosclerosis and connective tissue disorders are aetiological factors that are associated with ascending aortic aneurysms.^5^ Medical therapy or various surgical interventions may reduce the risk factors.

Diameter, connective tissue disease (e.g. Marfan or Loeys– Dietz syndrome), pregnancy, bicuspid aortic valve (BAV), familial history of thoracic aortic aneurysm and dissection, hypertension, gender, and aortic growth are factors that may influence the need for surgery.^5^ Size of the aneurysm is considered the most important independent factor in the decision for a patient to undergo surgery.

The required indications for RAA are acute dissection, rupture and intramural haematoma.^6^ Elective indications are generally prophylactic in nature, and they aim to prevent progression of aortic insufficiency and aortic rupture or dissection.

Aortic aneurysms are usually asymptomatic, with slow growth and they may develop distal thromboembolism, rapid expansion and rupture, with catastrophic complications. The law of Laplace predicts that, as the aneurysm size increases, wall tension also rises.^7^

Dissections have a high early mortality rate of up to 1–2% per hour. In patients with atherosclerotic aneurysms of the ascending aorta, rupture is the most common cause of death.^8^ Joyce et al. (1964) found that approximately 50% of patients with thoracic aortic aneurysms died within five years of the diagnosis.^9^

Aortic surgeries are complex and have high morbidity and mortality rates. The risks of surgery include paraplegia, stroke, bleeding and death; however rupture, dissection and death may also occur when the aneurysm is left unoperated. If no intervention is done at the time of cardiac surgery, the aneurysm may develop and rupture, and will need a second open-heart procedure, with additional technical challenges and complications. An initial concomitant surgery may increase the operative risks but protects patients from long-term aneurysmatic complications.

Replacement of ascending aortic aneurysms often requires significant additional surgery, such as CABG and AVR. Severe aortic valve disease is occasionally associated with dilation of the ascending aorta. One possible cause of dilation is haemodynamic flow disturbance in the aorta beyond the stenotic valve. The second possibility is a genetic predisposition to aortic dilation.

We performed AVR and RAA in 36 patients and Bentall in 11 patients. In recent years, ascending aortic surgery has been performed in an increasingly elderly population, with a 15–40% incidence of co-existing CAD. Therefore pre-operative coronary angiography is routinely performed in elective patients, and concomitant CABG has begun to be performed more frequently, with an incidence of between 11 and 25% in some larger aortic surgery studies.^10^,^12^ We performed CABG and RAA in 25 patients, and eight underwent both CABG and AVR with RAA. In our study it was shown that the aneurysm was discovered in patients who underwent coronary angiography for symptoms of ischaemia.

Ueda et al. identified incomplete coronary revascularisation as a risk factor for cardiovascular events.^13^ Atherosclerosis and inflammation are important factors in the development of valve stenosis and CAD.^14^,^15^ CAD is also a common finding in patients undergoing endovascular or surgical repair of descending, thoraco-abdominal or abdominal aortic aneurysms.^16^,^17^ In older patients undergoing planned aortic reconstruction, pre-operative coronary angiography should be performed and appropriate revascularisation must be performed.^18^. We suggest that surgery of the ascending aorta with concomitant CABG may increase the mortality rate.

A study in the UK showed that the overall mortality rate was 3.2% for isolated aortic valve procedures and 6.8% for aortic valve procedures with concomitant CABG.^10^ However, Ueda et al. reported that complete revascularisation of major coronary arteries with significant stenosis is essential to reduce postoperative cardiac events.^13^

In a report by Houel and colleagues from France, the type of surgery had no effect on long-term survival, but AVR + RAA was associated with more aortic wall complications (aortic root dilation and false aneurysms) than the Bentall procedure. However, AVR + RAA was performed in patients with Marfan syndrome and others with aortic root aneurysm.^19^

Yun and colleagues compared 255 patients who underwent AVR + RAA, and 135 patients who underwent the Bentall procedure between 1965 and 1995.^20^ In the AVR + RAA group, the surgical mortality rate was 15.3%. Survival at 10 years was 51 ± 3% and at 15 years it was 36 ± 3%. Urbanski and colleagues reported a similar operative risk and late mortality and morbidity among 100 patients who underwent AVR + RAA or a modified Bentall procedure using Carbomedics mechanical valves.^21^

Sioris and associates reported no differences in peri-operative mortality rate or freedom from re-operation in 133 patients after 10 years between AVR associated with RAA and a modified Bentall procedure.^22^ Rizzoli and colleagues, in their study of 809 patients undergoing AVR, including 110 RAA patients, reported a 30-day mortality rate of 5.5%.^23^ Garrido-Olivares et al. reported on combined AVR and supracoronary RAA in 89 patients with an operative mortality rate of 2.3%.^24^

Simple AVR does not prevent the enlargement of the ascending aorta. Patients who have dilated ascending aortas at the time of AVR are at high risk of developing postoperative ascending aortic complications.^25^ This is not because of the primary surgery but due to intrinsic changes in the aortic wall. The time interval between initial AVR and late ascending aortic events ranged from two to 18 years. In a study by Tsutsumi et al., 50% of patients who developed late ascending aortic events during the follow-up period died.^26^

Replacement of the ascending aorta does not significantly increase the mortality risk. Moreover, AVR cannot reduce the risk of fatal aortic complications. Some authors reported that in patients with bicuspid aortic valve after AVR only, the aorta continues to enlarge and aorta-related complications increase.^27^,^28^ It was shown that concomitant RAA during AVR did not increase the rate of morbidity and mortality in the short-term, despite an increase in aortic cross-clamp and total cardiopulmonary bypass times.^29^ Our findings are compatible with these studies.

The goal should be to avoid the catastrophic consequences of acute aortic dissection or rupture. The decision for surgical treatment is primarily based on comparative estimation of the natural prognosis of the disease versus the prognosis with treatment.^30^ The spontaneous prognosis is related to aortic diameter, mechanical properties of the vascular wall, and blood pressure. Aortic diameter has been best studied and is considered the primary prognostic parameter.30 The prognosis after treatment depends on complications of surgery and its mortality risk. In our study the mortality rate was increased insignificiantly.

Type I aortic dissection occurs in 0.6% of patients late after AVR.^31^ Thirteen per cent of patients with acute type I aortic dissections had a history of previous AVR.^32^ In contrast to previous AVR, a history of CABG alone is not an independent risk factor for type I aortic dissection.^33^ However, diameter is not specific enough to affect the risk of dissection, and the law of Laplace must be taken into account; the incidence of dissection and rupture increases with increasing size of the ascending aortic aneurysm.^34^ The primary aim of prophylactic replacement is to prevent this catastrophic complication.

Our decision to replace the ascending aorta was based on the size of the aortic diameter. Patients with ascending aortic diameter more than 45 mm underwent concomitant ascending aorta replacement.

In their study, Davies at al. reported that relative aortic size is more important than absolute aortic size in predicting complications. A new measurement, the aortic size index, which takes into account both aortic diameter and body surface area, was used for calculating the risk of negative events. According to them, increasing aortic size index was a significant predictor of increasing rates of rupture.^35^

Lentini at al. reported their initial experience for ascending aortic surgery with or without valve or root surgery via a ministernotomy approach. Surgery of the aortic root and ascending aorta has traditionally been performed via a conventional median full sternotomy. The development of minimally invasive surgical techniques reduces surgical trauma, length of mechanical ventilation and ICU stay, improves post-operative outcomes and also has cosmetic benefits.^36^

Especially in high-risk patients, a mini-sternotomy approach can improve recovery of respiratory function and allow earlier extubation, reducing ICU and hospital stay in complex aortic surgery. There are a few examples of this procedure: Tabata et al.^37^ and Perrotta et al.^38^ reported on a Bentall procedure with mini-sternotomy, using this approach on both elective and emergency patients, and in redo surgery. Svensson et al. reported 36 patients operated for ascending aorta replacement, of whom 18 had aortic arch repair through mini-sternotomy.^39^ The minimal incision does not allow for easy manipulation of the aortic arch while using selective antegrade cerebral perfusion. The need for CABG was the exclusion criterion in our study.

There are several ways of dealing with an enlarged aorta. One is by remodelling the aorta, using a supracoronary graft, or with a valved conduit. Some surgeons^40^ suggest a conservative approach and would rather remodel the aorta but we believe that the risk of recurrent dilatation of the aorta remains, so a more radical approach of supracoronary graft replacement is the main choice in our clinic. We use hypothermic circulatory arrest and antegrade cerebral perfusion generally, as we have more experience with this approach. We do not have experience of the minimal approach.

Some authors have argued against the strategy of routinely replacing the enlarged ascending aorta, based on their observations that significant increase in the ascending aorta after simple AVR is infrequent.^41^ We have not experienced this. The results of our study suggested that concomitant RAA did not significantly increase the mortality rate despite an increase in the aortic cross-clamp and total cardiopulmonary bypass times.

If type A aortic dissection is life threating but elective surgery carries a low surgical risk, we prefer to perform surgery earlier. According to the Society of Thoracic Surgeons, the operative risk associated with replacement of the proximal aorta under elective circumstances is 3.4%.^42^

In our study, the mortality rate was 5.3% in patients who underwent RAA alone, 11.1% in those who underwent AVR, 20% in those who underwent CABG, 37.5% in patients who underwent AVR + CABG, and 18.2% in those who underwent the Bentall procedure. We found no statistically significant differences between mortality rates. Therefore, this study supports the strategy of concomitantly replacing the ascending aorta at the time of cardiac surgery to prevent possible aortic rupture, dissection or death.

## Study limitations

This was a retrospective, single-centre, observational study and it had a small patient population. These results were also obtained in a small-volume centre (< 40 aortic operations per year), therefore the results cannot be generalised. In addition, we examined data from surgeries that were performed by different surgical groups, which limits standardisation. This study reflects clinical experiences in a small area and cannot be generalised for all hospitals. Prospective studies are required to define optimal prophylactic surgical techniques.

## Conclusions

It is common to deal with an associated ascending aortic aneurysm during concomitant surgery. In this retrospective study, we investigated whether concomitant surgery was a predictor for mortality after proximal aortic replacement. We found that the procedure for simultaneous correction of the ascending aorta and concomitant cardiac surgery (CABG, AVR, Bentall procedure) could be performed safely and with good results. Our study supports replacement of the ascending aorta during AVR or CABG if the aortic dimension is larger than 45 mm, to prevent aortic dissection or rupture after the procedure. As we have become more successful in performing the surgery, a higher proportion of patients undergoing open-heart surgery and requiring RAA will be operated on. A multicentre study is necessary to develop the best strategy for treatment of these patients.
